# The 16S rRNA gene sequencing of gut microbiota in pigeons infected with pigeon paramyxovirus type 1

**DOI:** 10.3389/fcimb.2026.1796212

**Published:** 2026-05-13

**Authors:** Xiaolong Gao, Qingsong Han, Kaiyu Zhu, Lina Tong, Jun Kui, Yongliang Chao

**Affiliations:** 1 College of Agriculture and Animal Husbandry, Qinghai University, Xining, China; 2 College of Animal Science, Wenzhou Vocational College of Science and Technology, Wenzhou, China; 3 Xining Animal Disease Prevention and Control Center, Xining, China; 4 Haidong Animal Disease Prevention and Control Center, Haidong, China

**Keywords:** 16S rRNA gene, gut microbiota, intestinal pathogenesis, pigeon, PPMV-1 infection

## Abstract

Pigeon paramyxovirus type 1 (PPMV-1) infection induces gastrointestinal symptoms, including diarrhea, and causes severe histopathological damage in the intestinal tract of pigeons. However, its impact on the gut microbiota of pigeons remains largely unknown. In this study, ten 4-week-old healthy racing pigeon squabs were randomly divided into a PBS group and a QH group. This two groups were respectively inoculated with PBS and PPMV-1/QH-01/CH/23 strain via the intraocular and intranasal routes. Cecal contents were subsequently collected for 16S rRNA gene sequencing. The results revealed that PPMV-1 infection did not significantly affect the richness and diversity of the gut microbiota but restructured its composition. The gut microbiota of pigeons were dominated by the phyla *Firmicutes*, *Proteobacteria*, and *Actinobacteriota* in both groups. However, the abundance of *Bacteroidota* was significantly reduced in the QH group. Furthermore, the QH group showed a significant increase in the abundance of *Clostridium_sensu_stricto_1*, particularly the pathogenic species *Clostridium paraputrificum* and *Clostridium perfringens*. In contrast, the abundances of commensals such as *Enterococcus* (especially the beneficial *Enterococcus columbae*) and *Mycoplasma* (specifically *Mesomycoplasma moatsii*) were significantly decreased. These findings indicate that PPMV-1 infection restructures the pigeon gut microbiota, offering valuable insights into the intestinal pathogenesis of PPMV-1.

## 1 Introduction

The gastrointestinal tracts (GIT) of humans and animals harbors an immense and diverse community of microorganisms, collectively forming a “superorganisms” with the host (El Aidy et al., 2015). These microorganisms inhabited in the gut contributes to the host health through multiple aspects. It helps maintain the structural and functional integrity of the gastrointestinal tract, which is essential for nutrient digestion and absorption. Furthermore, gut microbiota directly or indirectly participate in various host metabolism processes. They also play a crucial role in immune system development and function, modulating host immune response (de Vos et al., 2022). In addition, the gut microbiota is implicated in the development of various diseases, including gastrointestinal, metabolic, neurological, autoimmune, and infectious diseases (Qiu et al., 2022; Wang et al., 2025; Yao et al., 2024; Yuan et al., 2023; Zhao et al., 2022). In the context of viral infection, for example, comparative analyses reveal that the fecal microbiome of COVID-19 patients exhibits a marked reduction in diversity, characterized by a depletion of short-chain fatty acid-producing bacteria and an enrichment of opportunistic pathogens from the *Enterobacteriaceae* family, relative to the healthy individuals (Zhang et al., 2023). In H9N2 AIV-infected chickens, dysbiosis of the commensal gut microbiota was also observed, characterized by an increased relative abundance of pathogenic *Proteobacteria*, including *Shigella*, along with a concomitant reduction in the number of lactic acid-producing bacteria (Oakley and Kogut, 2016). Similarly, significant alterations in gut microbial composition have been reported in HBV patients, marked by an enrichment of pathogenic taxa such as *Firmicutes, Prevotella, Proteobacteria, and Streptococcus* (Yun et al., 2019; Chen et al., 2011). Furthermore, enteric viruses, including rotavirus (RV), norovirus, adenovirus, and astroviru, which in turn can reshape the human gut microbiome by promoting a shift in the dominant phylum from *Bacteroidetes* to *Firmicutes*, reducing microbial diversity, and increasing the abundance of opportunistic pathogens such as *Shigella* (Ma et al., 2011). Collectively, these findings suggest that infection-induced dysbiosis is a common phenomenon across different host species and pathogens. In line with this, Newcastle disease virus (NDV) infection has been shown to induce profound disruptions in the gut microbiota of chickens, with the magnitude of these effects varying significantly across different intestinal segments (Cui et al., 2018). Building on these observations, our research further demonstrates that the virulence of the NDV strain serves as a critical determinant, as strains with high and low virulence exert distinct and differential impacts on the composition and diversity of the chicken gut microbiota (Tong et al., 2022).

Pigeon paramyxovirus type 1 (PPMV-1), a variant of avian NDV genotype VI that has adapted to pigeons, is the causative agent of pigeon ND. This disease is characterized by high morbidity and mortality, especially in squabs (Zhan et al., 2022). Infected pigeons predominantly exhibit neurological signs, as well as gastrointestinal symptoms such as diarrhea (Zhang et al., 2022). However, the influence of PPMV-1 infection on the gut microbiota of pigeons remains unexplored. Qinghai constitutes a critical node within the global Central Asian Flyway for migratory birds (Li et al., 2020; Tian et al., 2024), playing a pivotal role in the ecology of NDV transmission. Recently, our laboratory isolated a PPMV-1 strain designated PPMV-1/QH-01/CH/23 (QH-01) from the Qinghai-Tibet Plateau, which has been demonstrated to be highly pathogenic in both chickens and pigeons (Tong et al., 2024). The extensive movement range of pigeons further increases the exposure risk of other wild birds and chickens to this isolate. Therefore, a comprehensive understanding of the pathogenesis of PPMV-1 from multiple perspectives is necessary.

In the present study, four-week-old healthy pigeon squabs were infected with the QH-01 strain to investigate the influence of PPMV-1 on gut microbiota by 16S rRNA gene sequencing technology. To our knowledge, this is the first report that illustrates the impact of PPMV-1 on pigeon gut microbiota.

## 2 Materials and methods

### 2.1 Viruses

QH-01 strain used in this study was propagated in the allantoic cavity of 9-day-old specific-pathogen-free (SPF) chicken embryos. The harvested allantoic fluid was stored at -70°C after determining the EID_50_. QH-01 strain was isolated from a sick racing pigeon in the Qinghai-Tibet Plateau, China in 2023. The mean death time of chicken embryos and the intracerebral pathogenicity index (ICPI) were 76.8 h and 1.25, indicating a mesogenic strain. Pigeon morbidity and mortality were 100% and 80%, respectively, therefore, this isolate was velogenic for both pigeons (Tong et al., 2024).

### 2.2 Animal Experiment

Ten healthy 4-week-old racing pigeon squabs without NDV HI antibodies were selected with similar body weights. In order to collect cecal contents from pigeons at the peak of clinical manifestation, the challenge virus dose and the sampling timepoint were determined based on the results of preliminary experiments. They were randomly divided into two groups with 5 pigeons per group, which were oculonasally inoculated with 0.1 mL PBS (PBS group) and 10^5^ EID_50_ (0.1 mL) of QH-01 strain (QH group), respectively. At 4 day post-challenge, pigeons were euthanized and cecal contents were collected aseptically. These samples were quickly frozen with liquid nitrogen and then stored in a refrigerator at -80°C for further research. All experimental procedures involving animals were approved and supervised by the Ethics Committee for the Care and Use of Laboratory Animals in Qinghai University, China (No. SL-2023048).

### 2.3 Bacterial 16S rRNA sequencing and bioinformatic analysis

Total genomic DNA was isolated from 200 mg of collected feces using the QIAamp 96 PowerFecal QIAcube HT kit (QIAGEN). Subsequently, the V3-V4 variable regions of the 16S rRNA gene were amplified from the genomic DNA using universal primers (343F 5′-TACGGRAGGCAGCAG-3′and 798R 5′-AGGGTATCTAATCCT-3′, product size is 455 bp). PCR amplification was performed according to the following system: 2×Gflex PCR buffer 20 μL, primer 343F (5 pmol/μL) 1 μL, primer 798R (5 pmol/μL) 1 μL, Tks Gflex DNA Polymerase 1 μL, and 50 ng DNA template. PCR amplification was performed under the following conditions: an initial denaturation step at 94 °C for 5 min, followed by 26 cycles consisting of three sequential steps-denaturation at 94 °C for 30 s, annealing at 56 °C for 30 s, and extension at 72 °C for 20 s. A final extension phase was then carried out at 72 °C for 5 min to ensure complete product synthesis. Subsequently, the PCR products were first visualized via gel electrophoresis to confirm amplification success, and then the 455 bp target band was purified using AMPure XP beads (Agencourt) to remove impurities such as unincorporated primers and dNTPs. The purified first-round PCR product served as the template for a second-round PCR, which employed index primer pairs (adapter I5 primer and adapter I7 primer) to introduce sample-specific barcodes. The reaction system for the second-round PCR was identical to that of the first round; the only modification was a reduction in the number of amplification cycles to 7. After a second purification step with AMPure XP beads, the final amplicons were quantified using the Qubit dsDNA Assay Kit to ensure consistent concentration across samples. Equal amounts of the purified amplicons were then sent to Shanghai OE Biotech Co., Ltd. for subsequent sequencing by Illumina MiSeq system.

Raw sequencing data were provided in FASTQ file format. Paired-end reads were then preprocessed using cutadapt software to detect and cut off the adapter. After trimming, paired-end reads were filtering low quality sequences, denoised, merged and detect and cut off the chimera reads using DADA2 with the default parameters of QIIME2. At last, the software output the representative reads and the ASV abundance table. The representative read of each ASV was selected using QIIME 2 package. The number of tags classified to each ASV in individual samples was counted to determine the abundance of each ASV across samples. Based on the ASV clustering results and research requirements, the number of core and unique ASVs among different samples (groups) was analyzed and visualized using a flower or Venn plot. Microbial diversity was analyzed in terms of alpha and beta diversity by QIIME2 software. Alpha diversity was estimated using the Chao1, Shannon and Simpson indices, while beta diversity was evaluated by principal coordinates analysis (PCoA) of Bray-Curtis distances calculated with the R package. Statistical comparisons between groups were conducted using Wilcoxon rank-sum test and Student’s t-tests, with a *p*-value < 0.05 considered statistically significant. In the present study, all sequences have been deposited to the National Center for Biotechnology Information (NCBI) database under accession number PRJNA1347967.

## 3 Results

### 3.1 Clinical symptoms of infected pigeons

Pigeons in the PBS group did not exhibit any clinical symptoms until euthanasia, whereas those in the QH group showed various clinical signs, such as diarrhea, breathing difficulties, paralysis, wing drop, incoordination and prostration, but no mortality occurred.

### 3.2 Sequencing results overview

Ten pigeon cecal content samples were collected from the QH group and the PBS group for 16S rRNA gene sequencing and analysis. After quality control, the data volume of clean tags ranges from 74,801 to 78,983. Valid (non-chimeric) tags per sample were distributed in the range of 71,800–77,216, and ASV counts varied from 27 to 78 across the samples. Importantly, Good’s coverage for all samples ranged from 99.99% to 100%, indicating that the sequencing depth was sufficient to encompass the majority of the gut microbiota in each sample.

### 3.3 Alpha diversity analysis and PCoA

**Figure 1:**
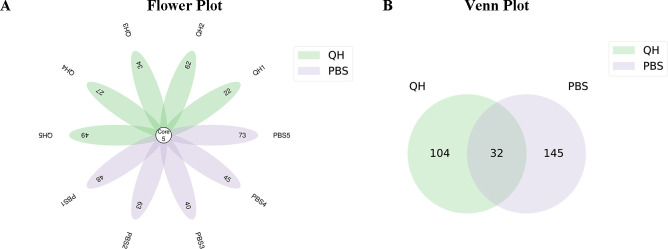
Analysis of ASVs in the two groups. **(A)** flower plot showing overlap in ASVs of differential abundance of each sample in QH and PBS groups. **(B)** Venn plot showing overlap in ASVs of differential abundance in QH and PBS groups.

Flower and Venn diagrams illustrates the core and unique ASVs across different groups. The number of core ASVs in each sample was 5. The number of ASVs shared by the two groups was 32, while the number of ASVs unique to the QH group was 104, which was lower than the 145 of the PBS group, indicating that viral infection reduced the species richness of the gut microbiota in pigeons (**Figure 1**).

**Figure 2:**
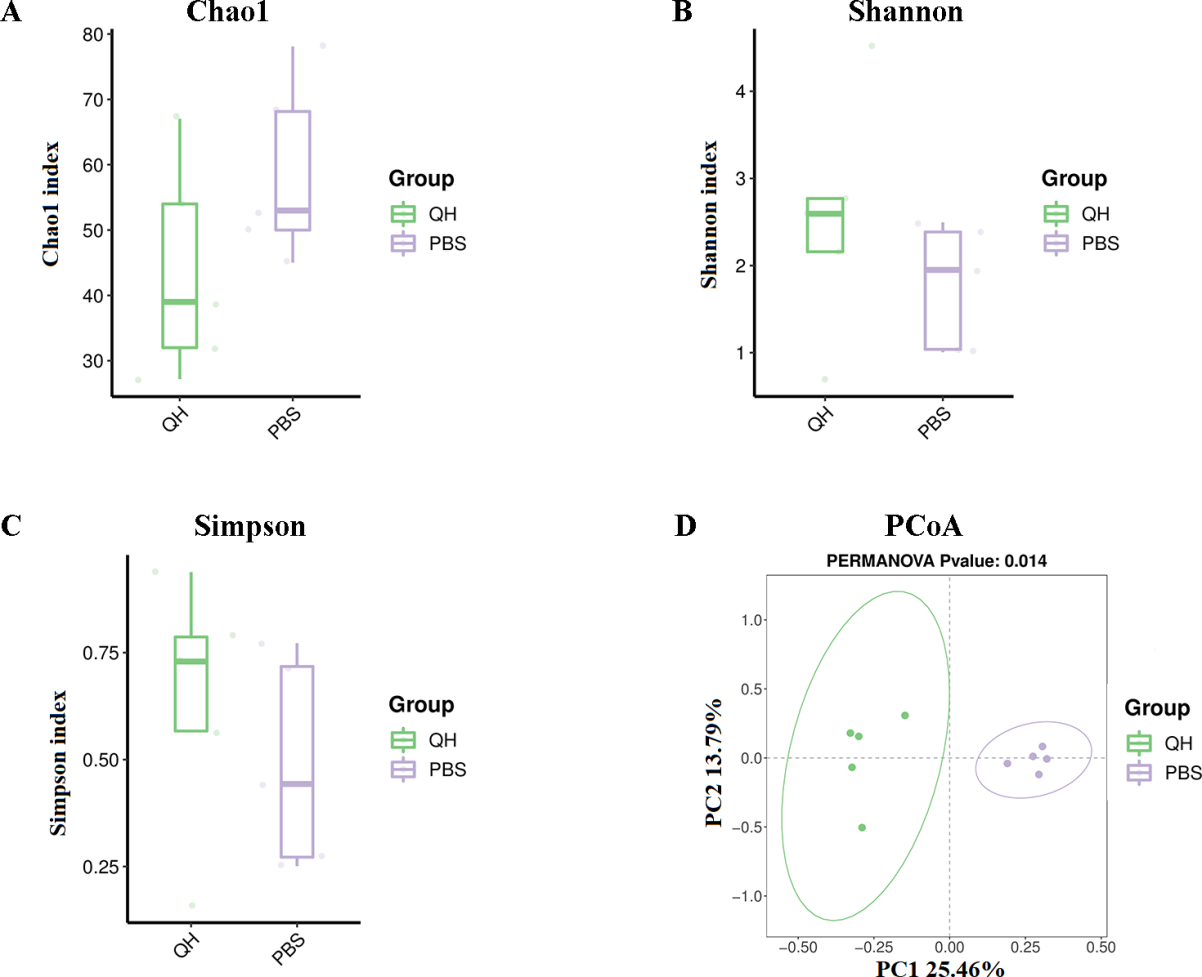
The microbial diversity and richness index analysis. **(A)** Chao1 index. **(B)** Shannon index. **(C)** Simpson index. **(D)** PCoA of QH and PBS groups.

To assess the influence of PPMV-1 infection on gut microbial diversity and richness, the Chao, Shannon, and Simpson indices were calculated and presented for each sample in **Table 1**. Among the alpha diversity indices, the Chao1 index can reflect the relative abundance of community distribution, while the Shannon and Simpson indices can comprehensively reflect species richness and evenness. As shown in **Figure 2A**, the average Chao1 index of the QH group was 43.844 and the PBS group was 58.857. The average Shannon indices of the QH and PBS groups were 2.547 and 1.775, respectively (**Figure 2B**). The average Simpson indices of the QH and PBS groups were 0.6357 and 0.4911, respectively (**Figure 2C**). A series of pairwise t tests (using non-pooled standard deviations) indicated that there were no significant differences among the Chao, Shannon, and Simpson indices (p > 0.05), implying that bacterial richness and diversity remained unaffected by PPMV-1. PCoA was performed based on Binary-Jaccard distances to visualize the differences between individuals or groups. As shown in **Figure 2D**, the contributions of PC1 and PC2 to the total variation were 25.46% and 13.79% respectively (P = 0.014), and a clear clustering tendency can be observed for samples from each group. There was a significant separation between the two groups, indicating a significant difference in their microbial communities (p = 0.014).

**Table 1:** The alpha estimator summary.

Sample	PD_whole_tree	Chao1	Shannon	Simpson	Observed species	Goods coverage	ACE
QH1	2.3456	27.2	0.689099	0.157740	27	0.999975	25
QH2	6.5867	67.02	4.525550	0.938063	67	0.999996	67
QH3	3.5566	39	2.769148	0.786684	39	0.999994	38
QH4	3.9115	32	2.594840	0.729223	32	0.999999	32.383276
QH5	5.1355	54	2.157722	0.566787	54	0.999994	54.377188
PBS1	5.4421	53	1.038288	0.271928	53	0.999996	53.536560
PBS2	7.1359	68.15	2.495535	0.717743	68	0.999994	67.616333
PBS3	6.0110	45.03	2.385549	0.771910	45	0.999992	45
PBS4	5.6951	50	1.006009	0.250895	50	1	50
PBS5	7.6721	78.1	1.949554	0.442994	78	0.999984	78.969246

### 3.4 Microbial composition analysis

**Figure 3:**
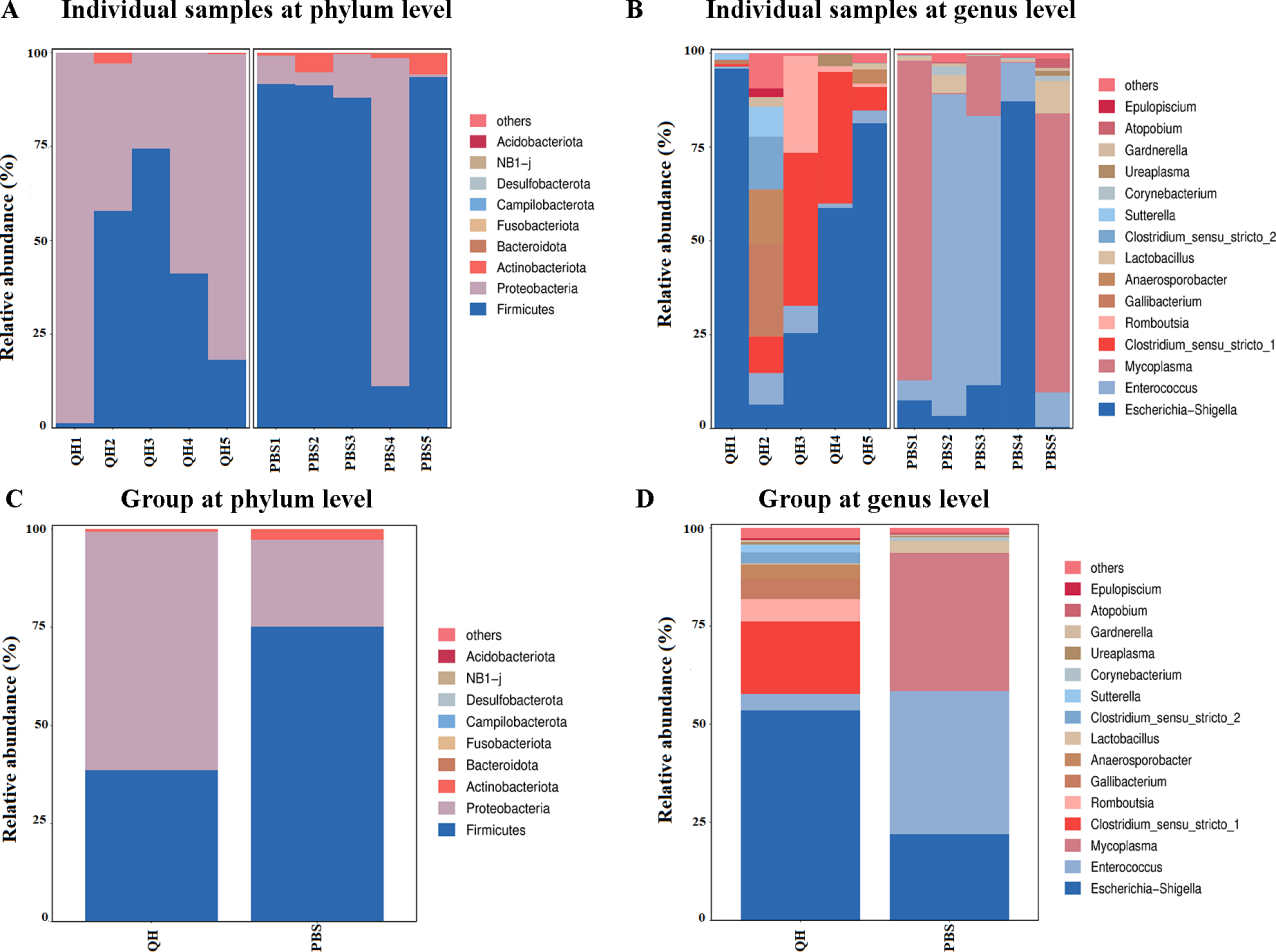
Microbial composition of different samples and groups. **(A)** taxa assignments of each sample at phylum level. **(B)** taxa assignments of each sample at genus level. **(C)** taxa assignments of each group at phylum level. **(D)** taxa assignments of each group at genus level.

To further elucidate the impact of PPMV-1 infection on the gut microbiota, its composition was assessed at various taxonomic levels. Although the relative abundances of microbes varied among individual samples at the phylum level, the overall composition was primarily dominated by Firmicutes, Proteobacteria, and Actinobacteriota in both groups (**Figures 3A, C**). At the genus level, the genera with relatively high abundance in the QH group were Escherichia-Shigella, Clostridium_sensu_stricto_1, and Romboutsia in sequence, while those in the PBS group were Enterococcus, Mycoplasma, and Escherichia-Shigella in sequence (**Figures 3B, D**). Moreover, genus Escherichia-Shigella accounted for a higher proportion in the QH group compared to the PBS group.

**Figure 4:**
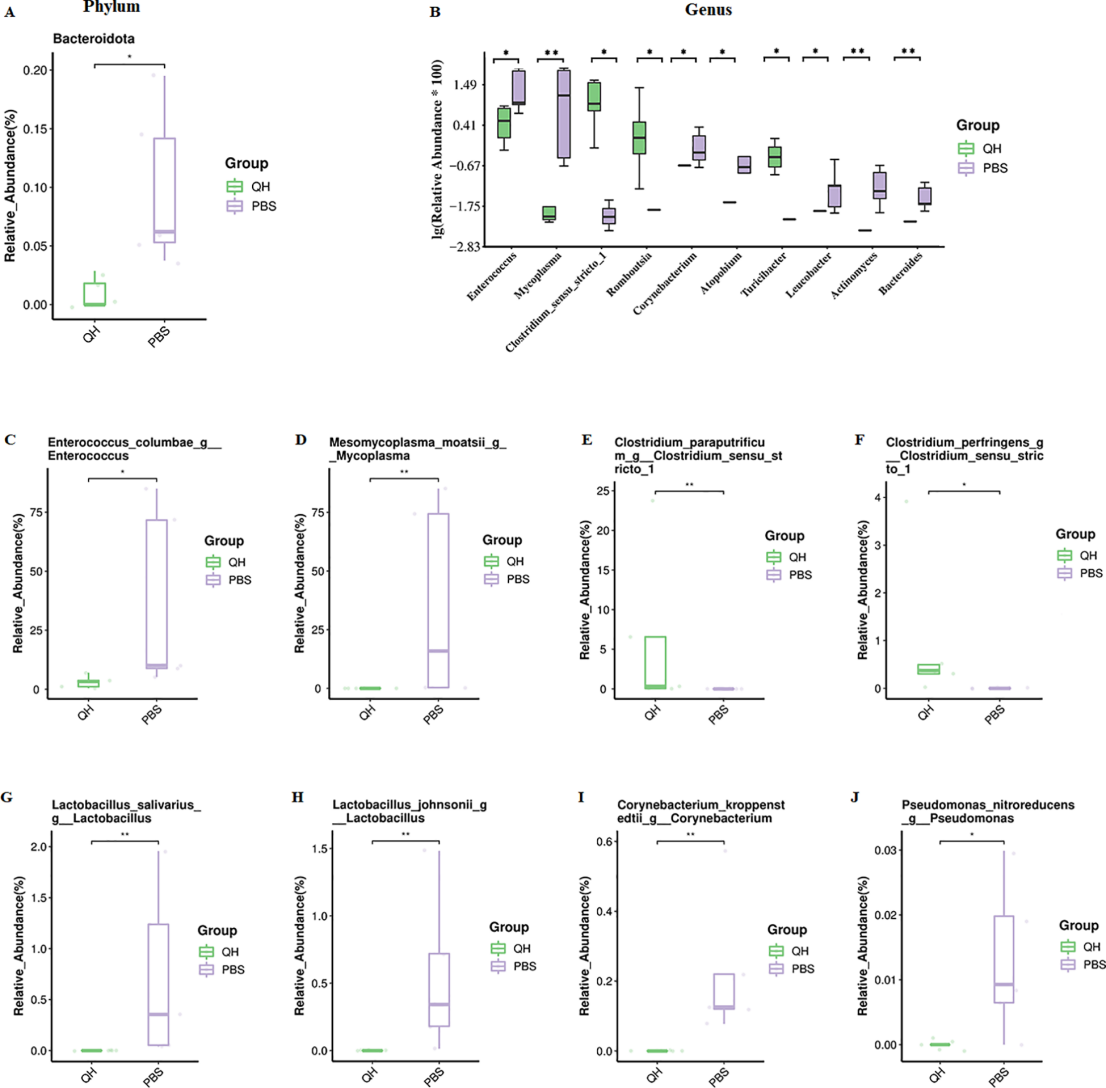
The relative abundance of the top 10 different bacteria in the two groups. **(A)** atphylum level; **(B)** at genus level; **(C–J)** at species level. *, P-value <0.05; **, 0.05<P-value ≤0.01.

Wilcoxon rank-sum test with FDR-adjusted analysis between the two groups revealed 1 differential phylum (**Figure 4A**), 11 differential genera (**Figure 4B**) and 8 differential species (**Figures 4C–J**). At the phylum level, the abundance of Bacteroidota decreased significantly in QH group in relation to that in the PBS group (p<0.05). At the genus level, the top ten differential genera were Enterococcus, Mycoplasma, Clostridium_sensu_stricto_1, Romboutsia, Corynebacterium, Atopobium, Turicibacter, Leucobacter, Actinomyces and Bacteroides. Among these, the relative abundances of Clostridium_sensu_stricto_1, Romboutsia, and Turicibacter, were significantly higher in the QH group compared to the PBS group, the others were more abundant in the PBS group than the QH group (p<0.05). At the species level, the relative abundances of Enterococcus_columbae_g:Enterococcus, Mesomycoplasma_moatsii_g:Mycoplasma, Lactobacillus_salivarius_g:Lactobacillus, Lactobacillus_johnsonii_g:Lactobacillus, Corynebacterium_kroppenstedtii_g:Corynebacterium and Pseudomonas_nitroreducens_g:Pseudomonas in QH group were significantly lower than those in PBS group (p<0.05), however Clostridium_paraputrificum_g:Clostridium_sensu_stricto_1 and Clostridium_perfringens_g:Clostridium_sensu_stricto_1 in QH group were significantly higher than those in PBS group (p<0.05).

## 4 Discussion

The GITs of humans and animals harbors a diverse and highly active microbiota, composed of bacteria, eukaryotes, archaea, and viruses. The diarrhea caused by enteric viral infections often result in bacterial dysbiosis or an imbalance of bacterial proportion within the GIT microbiota. Such dysbiosis can promote the overgrowth of opportunistic pathogens such as *Campylobacter*, *Neisseria*, and *Enterobacteriaceae*, which are associated with disease complications (Chen et al., 2017). Meanwhile, gut microbiota and their metabolites can also modulate the progression of viral infections via multiple mechanisms, such as direct interactions with virions, alteration of the gut microenvironment, and systemic regulation of innate and adaptive immunity (Campbell et al., 2023). For instance, specific bacterial species have been shown to bind coxsackievirus B3 and enhance its infectivity and stability in a mouse model (Dhalech et al., 2021). Similarly, microbial-derived butyrate may influence susceptibility to hand, foot and mouth disease (Guo et al., 2021). Moreover, by maintaining low-level constitutive expression of type I interferons (IFNs), the gut microbiota enables the rapid induction of antiviral activity upon infection (Schaupp et al., 2020). PPMV-1, the causative agent of pigeon ND, also causes diarrhea in infected pigeons. Nevertheless, its impact on the pigeon gut microbiota remains unclear. Therefore, this study employed 16S rRNA sequencing to investigate the structural changes in the gut microbiota of pigeons following PPMV-1 infection.

In the present study, cecal content samples were collected from pigeons in the PBS and QH groups for 16S rRNA sequencing, followed by intergroup comparative analysis. The results revealed no significant differences in the alpha diversity indices (Chao1, Shannon, and Simpson) between the two groups, indicating that QH-01 infection does not affect the richness and diversity of the pigeon gut microbiota. However, PCoA demonstrated a clear separation between the two groups, suggesting that QH-01 infection significantly altered the structural composition of the pigeon gut microbiota. These findings are consistent with previous reports on the effects of NDV infection on the richness, diversity, and composition of the cecal microbiota in chickens (Cui et al., 2018; Tong et al., 2022). In our study, the cecal microbiota of healthy pigeon squabs in the PBS group was dominated by the phyla *Firmicutes*, *Proteobacteria*, and *Actinobacteriota*, which is consistent with the observations of Ji et al (Ji et al., 2020). In contrast, QH-01 infection led to a marked reduction in the abundance of *Enterococcus* (especially *Enterococcus_columbae*), and *Mycoplasma* (especially *Mesomycoplasma_moatsii*), while significantly increasing the abundance of *Clostridium_sensu_stricto_1* (especially *Clostridium_paraputrificum* and *Clostridium_perfringens*) (*p*<0.05). The observed gut microbiota dysbiosis following PPMV infection (e.g., increased abundance of *Clostridium* spp. and decreased abundance of *Enterococcus columbae*) may result from either the direct effects of viral-microbiota interactions or indirect consequences of infection, such as reduced feed intake, dehydration, or host immune responses.

*Enterococcus columbae* is a host-specific bacterium first isolated from pigeons in 1990 (Butaye et al., 2002; Devriese et al., 1990). Most *E. columbae* isolates exhibit alkaline phosphatase activity and ferment trehalose, whereas only a minority show arginine dihydrolase and pyroglutamic acid arylamidase activities, or the ability to metabolize glycogen (Dolka et al., 2020). Due to its ability to produce antimicrobial peptides, *E. columbae* can be beneficial to pigeons under certain circumstances.

(Martin et al., 2006). They also appear to pose a low health risk, given the scarcity of potential virulence genes and their susceptibility to most clinically relevant antibiotics (Martin et al., 2006). *E. columbae* dominates the gut microbiota of racing pigeons, with its abundance varying throughout the race season. Our observation of a high abundance of *E. columbae* in healthy pigeons further supports these earlier findings. In contrast, QH-01 infection resulted in a marked decrease in its abundance. This indicates that PPMV-1 infection causes dysbiosis of these enterocin-producing bacteria in the gut; nevertheless, the specific functions and the involvement of this bacterium in the pathogenesis of PPMV-1 requires further clarification.

*Mycoplasma moatsii* was first reported in 1974 from healthy grivit monkeys and in 1990 *M. moatsii* was isolated from the intestine of the progeny of wild Norway rats (Giebel et al., 1990). Klostermann and Lierz isolated *M. moatsii* from the choana of a barn swallow without any clinical signs (Klostermann and Lierz, 2023). In our study, 16S rRNA sequencing revealed the presence of *M. moatsii* in the pigeon intestine. The abundance of *M. moatsii* was significantly higher in healthy pigeons than in QH-01-infected pigeons, suggesting it is more likely a commensal bacterium, although its precise function remains unclear. Given that mycoplasmas commonly exist in the environment and can readily contaminate samples during experimental procedures, the results obtained require further validation in subsequent experiments using methods such as bacterial isolation and identification, as well as quantitative real-time PCR (qPCR).

In addition to the reduction in beneficial bacteria, a significant increase in pathogenic bacteria was observed. The genus *Clostridium*, comprising more than 180 species, represents one of the largest bacterial groups associated with bacteremia (Balas-Maftei et al., 2024). Among these, *Clostridium perfringens* is the most common species (42%), whereas *Clostridium paraputrificum* has been identified in only 1% of cases (Intra et al., 2020). Although *C. paraputrificum* is generally a harmless commensal in the intestinal flora, bacteremia caused by this organism typically occurs secondary to intestinal mucosal injury and in the presence of predisposing conditions such as gastrointestinal disorders, malignancies, diabetes, HIV infection, or neutropenia (Mostel et al., 2022; Pramana et al., 2025; Rodriguez et al., 2023). *C. perfringens* is a normal component of the gut microbiota in healthy chickens (Rana et al., 2023). However, dysregulation of the intestinal microbiota can trigger the proliferation of virulent *C. perfringens* strains, leading to massive toxin production and ultimately causing necrotic enteritis in chickens (Dierick et al., 2024). Several predisposing factors, including coccidial infections, immunosuppressive viruses (such as infectious bursal disease virus), and abrupt changes in dietary protein content, have been identified as contributors to the adhesion and colonization of *C. perfringens* in the intestinal mucosa (Gautam et al., 2024). In line with these previous reports, both *C. paraputrificum* and *C. perfringens* were significantly increased in pigeons infected with QH-01. The sustained microbial dysbiosis, together with the increased abundance of these pathogenic bacteria, likely contributes to the more severe gut symptoms observed in the QH group compared to the PBS group during viral infection. Due to the small sample size, the above findings have certain limitations. Subsequent bacterial isolation, identification, and correlation analysis will be performed to further elucidate the relationship between PPMV-1 infection and gut microbiota alterations.

Accumulating results from 16S rRNA gene sequencing studies indicates that avian viral infections consistently induce gut microbiota dysbiosis. A common feature across different viral infections, including NDV, avian influenza virus (AIV), infectious bronchitis virus (IBV), and duck enteritis virus (DEV), is a reduction in α-diversity and a significant shift in β-diversity (Cui et al., 2018; Chrzastek et al., 2021; Herath Mudiyanselage et al., 2025; Kong et al., 2026). At the phylum level, a decreased *Firmicutes/Bacteroidetes* ratio and an expansion of *Proteobacteria* are repeatedly observed, including our present study (Abd El-Hack et al., 2022). At the genus level, depletion of beneficial bacteria such as *Lactobacillus* and *Enterococcus* alongside overgrowth of opportunistic pathogens like *Escherichia-Shigella* and *Clostridium* spp. appears to be a conserved response (Tong et al., 2022; Chen et al., 2019). Furthermore, the gut microbiota plays a bidirectional role in modulating antiviral immunity, as antibiotic-induced dysbiosis increases susceptibility to viral infection, while a healthy microbiota enhances host resistance (Abd El-Hack et al., 2022; Figueroa et al., 2020; Yin et al., 2022). Despite these similarities, notable differences exist. Viral virulence is a key determinant: velogenic NDV strains induce profound phylum-level changes, whereas lentogenic strains cause only minor alterations (Tong et al., 2022). Host genetic background modulates the β-diversity response without altering the overall α-diversity trend (Chrzastek et al., 2021). Additionally, tissue-specific responses are evident, as IBV infection alters cecal and tracheal microbiomes with distinct time courses (Herath Mudiyanselage et al., 2025). The results of the present study are consistent with previous findings at the phylum level, but show both similarities and differences at the genus level. These observations collectively suggest that while core patterns of dysbiosis are conserved across avian viral infections, the specific outcomes are influenced by viral species, viral virulence, host genetics, infection stage, and anatomical site. Future studies using gnotobiotic models and multi-omics approaches are warranted to dissect the causal mechanisms underlying virus-microbiota interactions in birds.

In conclusion, our study demonstrated that PPMV-1 infection induced significant dysbiosis in the gut microbiota of pigeons. This alteration was characterized by a reduced relative abundance of the genera *Enterococcus* and *Mycoplasma*, and an increase of *Clostridium_sensu_stricto_1*. Therefore, prevention or mitigation of viral infection induced bacterial dysbiosis, such as the application of probiotics, prebiotics, or phage therapy, may be a promising future therapeutic avenue.

## Data Availability

The datasets presented in this study can be found in online repositories. The names of the repository/repositories and accession number(s) can be found in the article/supplementary material.
